# Prof. Scarpa's Improved Operation for Aneurism

**Published:** 1817-05

**Authors:** James Briggs

**Affiliations:** 30, Edgeware Road


*To the Editors, &c.*

GENTLEMEN,

I BEG leave to transmit to you, for the information of your readers, a communication received in a letter from Professor Scarpa, dated March 30th, the object of which is the improvement of Mr. Hunter's Operation for Aneurism.

I am, Sir,

Your obedient Servant,

JAMES BRIGGS.

30, *Edgeware Road,*
*April* 18.

 "During

"During the last year, I have made many experiments on animals after the manner of Jones, in order to determine the most useful method of tying the large arteries of the limbs. From these experiments I have drawn several useful results, which I shall shortly publish; but one of them has particularly arrested my attention, viz. that, on the fourth day from the operation, the ligature may be safely removed from the artery, as the cohesion and closure of the vessel is then sufficiently strong to resist the impetus of the blood; and, consequently, that it is not necessary, as is usual, to wait until the ulcerative process has loosened the ligature from the artery. From sixteen to twenty days of valuable time is thus lost, during its detachment, which might be much more advantageously employed in the treatment of the wound, freed from every extraneous body, and disposed to heal by the first intention.

"The first trial was made on a man, on account of a popliteal aneurism, three months ago. The ligature was removed from the femoral artery on the fourth day after the operation; and the result perfectly answered my expectation, the pulsation in the aneurism being no longer renewed. As soon as I have collected a sufficient number of facts similar to this, I design to give a full detailed account of them. I find, in performing the operation in this manner, that a small compress [cylinder] of cloth, imbued with ointment, interposed between the artery and ligature, is of great utility, and, indeed, indispensible, in rendering the removal of the ligature easy; whereas, when applied singly round the vessel, it is so imbedded between the internal and middle ruptured coats, as to make the division of it unusually difficult and dangerous. By this method, on the contrary, it may be readily and safely divided upon the compress.

*Pavia; March* 30, 1817."

---

We expect further information from this truly illustrious professor; but may, at present, be permitted to remark (as we did concerning the silken ligatures proposed by some English surgeons) that, after Mr. Hunter's operation for aneurism, and after a successful amputation on Mr. Allison's method, we have often seen complete union by the first intent, excepting where it was interrupted by the ligatures, the ends of which were permitted to hang out till the parts attached to the arteries were, in a few days, so loosened by suppuration as to be removed without violence. —EDIT.

